# Analyzing Clinical Parameters and Bacterial Profiles to Uncover the COPD Exacerbations: A Focus on Intensive Care Unit Challenges

**DOI:** 10.3390/medicina61040669

**Published:** 2025-04-05

**Authors:** Dragoș Huțanu, Hédi-Katalin Sárközi, Mara Andreea Vultur, Adrian-Horațiu Sabău, Iuliu Gabriel Cocuz, Corina Mărginean, Andra-Maria Chelemen, Corina Eugenia Budin

**Affiliations:** 1 Pulmonology Department, “George Emil Palade” University of Medicine Pharmacy, Science and Technology of Târgu Mureș, 540139 Târgu Mureș, Romania; dragos.hutanu@umfst.ro (D.H.); mara.vultur@umfst.ro (M.A.V.); 2 Pathophysiology Department, “George Emil Palade” University of Medicine, Pharmacy, Science and Technology of Târgu Mureș, 540139 Târgu Mureș, Romania; adrian-horatiu.sabau@umfst.ro (A.-H.S.); iuliu.cocuz@umfst.ro (I.G.C.); corina.budin@umfst.ro (C.E.B.); 3 Oncology and Palliative Care Department, “George Emil Palade” University of Medicine Pharmacy, Science and Technology of Târgu Mureș, 540139 Târgu Mureș, Romania; corina.marginean@umfst.ro; 4 Faculty of Medicine, “George Emil Palade” University of Medicine Pharmacy, Science and Technology of Târgu Mureș, 540139 Târgu Mureș, Romania; chelemen.andra-maria@stud18.umfst.ro

**Keywords:** chronic obstructive pulmonary disease, exacerbations, intensive care unit, bacterial infections, inflammation

## Abstract

*Background and Objectives:* Chronic obstructive pulmonary disease (COPD) poses a significant healthcare challenge worldwide, frequently leading to exacerbations necessitating intensive care unit admissions for potentially life-threatening complications. We aimed to investigate correlations between laboratory parameters, bacteriological agents, ventilation mode, and survival rates among COPD patients admitted to the ICU. *Materials and Methods:* Data were collected from the Pulmonology Department of Mures Clinical County Hospital, Romania, from 1 January 2022 to 30 October 2023. Eighty-four COPD patients required ICU transfer, except for concurrent SARS-CoV-2 infections. *Results:* Ventilation modes exhibited a significant correlation with specific bacteriological agents, orotracheal intubation being more prevalent in infections with *Acinetobacter baumanii*, *Staphylococcus aureus*, and *Streptococcus pneumoniae* (*p* < 0.001). Negative cultures were predominantly found in patients managed with non-invasive ventilation. Laboratory parameters revealed an association between elevated white blood cell counts and bacteriological superinfection, particularly with *Escherichia coli* (*p* < 0.001). Different bacteriological agents had different survival rates. Patients infected with *Acinetobacter baumanii* exhibited the highest mortality rate, while those with Staphylococcus aureus had the lowest (*p* < 0.01). *Conclusions:* The importance of prompt evaluation of laboratory parameters and bacteriological findings is underscored by these findings, particularly in ICU settings where ventilation and bacteriological profiles influence patient outcomes. The identification of elevated WBC counts is a marker of bacterial superinfection. The association between specific bacterial agents and ventilation modes highlights the importance of tailored treatment based on microbial profiles. These insights can be applied to refine treatment protocols and enhance survival rates in severe COPD exacerbations that require ICU management.

## 1. Introduction

Chronic obstructive pulmonary disease (COPD) is a common progressive lung disease marked by ongoing respiratory symptoms and airflow limitation due to airway and alveolar damage from long-term exposure to harmful particles or gases [1]. COPD is a significant cause of illness and death worldwide, affecting about 328 million individuals and causing over 3 million deaths annually, making it the third leading cause of death globally [2]. The disease imposes a heavy burden, with frequent exacerbations substantially increasing healthcare costs and patient morbidity [1,2]. The prevalence of risk factors like smoking and environmental pollution, and the aging population in many parts of the world, compound the global burden of COPD, making this pathology one of the most prevalent noncommunicable diseases. The data available in Eastern Europe on the real prevalence of COPD are incomplete; they are taken only from the registries of general practitioners, according to the World Health Organization’s Prevalence of Chronic Obstructive Pulmonary Disease report from 2024 [3]. According to the Legislative Portal of the Romanian Government (GHID 05/10/2010), Romania ranks third in Europe for the COPD mortality rate for men, after Hungary and Ireland, with 60 deaths per 100,000 inhabitants [4].

Standard treatments for COPD aim to improve quality of life, reduce symptoms, and prevent exacerbations [2]. Pharmacological therapy, smoking cessation, pulmonary rehabilitation, vaccinations, and oxygen therapy are useful strategies.

Increased dyspnea, sputum production, and wheezing are common clinical findings that suggest an infectious exacerbation of COPD [5]. Pneumonia is identified through clinical presentation, chest X-rays showing localized infiltrates, and microbiological testing. The clinical course features acute onset of fever, cough, and pleuritic chest pain [6]. The treatment involves bronchodilators, corticosteroids, and antibiotics targeting potential pathogens [5]. *Mycobacterium tuberculosis* hominis is the most common cause of tuberculosis, which is a chronic respiratory infection that can be confirmed by microscopic and cultural examination and is treated by specific antituberculosis drugs [6].

Patients with COPD associate pulmonary and extrapulmonary comorbidities—bronchiectasis, obstructive sleep apnea, lung cancer, cardiovascular diseases (heart failure, coronary disease, atrial fibrillation), metabolic disorders (diabetes mellitus, renal insufficiency, hyperuricemia, and anemia), psychiatric and neurological pathologies, and osteoporosis—decreasing quality of life and increasing the rate of hospital admissions [7].

COPD exacerbations, characterized by acute worsening of respiratory symptoms, often require hospitalization and can lead to ICU admissions due to severe respiratory distress and potentially life-threatening complications. Severe acute exacerbation of COPD (AECOPD) with hypercapnic respiratory failure is a life-threatening condition that requires either invasive or, more commonly, non-invasive mechanical ventilation in an intensive care unit or a respiratory intermediate care unit. AECOPD hospitalization is linked to poor prognosis and an increased risk of death [8].

Managing COPD exacerbations in the ICU is challenging due to the necessity of individualized treatment based on different ventilation modes, the risk of bacterial superinfections, and the need for respiratory rehabilitation during and after ICU admission [9,10,11,12,13,14].

Bacterial infections are crucial in COPD exacerbations, with common pathogens including *Streptococcus pneumoniae*, *Haemophilus influenzae*, and *Moraxella catarrhalis* [15,16,17]. However, ICU-admitted COPD patients are also at risk of infections with multidrug-resistant organisms like *Acinetobacter baumannii* and *Staphylococcus aureus*, making treatment difficult and negatively affecting survival rates. Identifying the bacterial profile and understanding its relationship with ventilation modes and laboratory parameters is essential for optimizing treatment and improving prognostic outcomes [18,19,20].

Standard laboratory parameters such as white blood cell (WBC) counts, C-reactive protein (CRP) levels, and procalcitonin (PCT) levels are used in clinical practice to monitor inflammation and infection status in COPD patients. Elevated WBC counts, particularly, are associated with bacterial overgrowth and worse clinical outcomes. Prompt evaluation of these laboratory markers and bacteriological findings provides valuable insights for early intervention and appropriate management in the ICU [21,22,23].

## 2. Materials and Methods

This retrospective study explores potential correlations between laboratory parameters, bacteriological pathogen agents, ventilation mode, and survival rates among COPD patients admitted to the ICU. Specifically, by analyzing data from the Pulmonology Department at Mures Clinical County Hospital in Romania, we aim to identify potential correlations between these factors and explore how they influence patient outcomes during and after ICU admission.

We conducted a retrospective study over a 22-month period (1 January 2022–30 October 2023), including 84 patients who experienced acute exacerbations of chronic respiratory failure caused by COPD. The initial lot included 94 patients, but after applying the inclusion and exclusion conditions, the final sample consisted of 84 patients.

### Inclusion and Exclusion Criteria

Inclusion criteria:

- Patients aged 45 years or older
- Patients diagnosed with COPD according to GOLD 2024 criteria [2] (Global Initiative for Chronic Obstructive Lung Disease 2024)
- Regularly monitoring by pulmonologists (at least once every 3 months)
- At least one moderate or severe documented exacerbation in the last year
- Patients presenting clinical signs for an AECOPD (such as purulent sputum, elevated body temperature, and increased respiratory rate) and laboratory parameters (elevated white blood cell count, C-reactive protein) indicative of bacterial superinfection
- Patients admitted to the ICU for an acute exacerbation of chronic respiratory failure that required mechanical ventilation or non-invasive ventilation

Exclusion criteria:

- Patients admitted outside the study period
- Patients with a secondary diagnosis of SARS-CoV-2 infection
- Patients with overlap syndrome between bronchial asthma and COPD
- Patients who were not experiencing severe clinical deterioration and did not require ICU transfer

Data were collected retrospectively from the specified period through the hospital’s informatics system (Hipocrate version H3 concept), using general clinical observation sheets, discharge notes, and summaries from the included patients’ Intensive Care Units (ICU), adhering to confidentiality agreements.

Patients were admitted to the Pneumology Department from the Emergency Service. At admission, they usually present themselves with numerous investigations, such as chest radiography or CT scans, typical biochemical analyses, blood gas analyses (ABG), and quantitative determination of inflammatory markers.

In the Pneumology Department, various respiratory tract samples were collected from spontaneously expelled sputum following a deep cough or bronchial aspirations obtained through bronchoscopy. Blood cultures were drawn from the peripheral vein and collected using sterile containers. The samples were sent to the Microbiology Department, where they were processed according to standard microbiological techniques for bacteriological examinations to identify both aerobic and anaerobic pathogenic agents as well as fungi through direct microscopic analysis after Gram staining, in addition to in vitro culturing using blood agar and Sabouraud media. The initial examination occurred after overnight incubation at 37 °C; the samples were reincubated and reexamined after 48 h. If the clinical and radiological context highly suggests TB infection, we performed specific bacteriological investigations using Ziehl–Neelsen staining and Löwenstein–Jensen culture medium.

If the patient’s general condition deteriorated during admission to the Pneumology Department, characterized by an increased level of dyspnea, fatigue, and clinical signs of hypercapnic encephalopathy, we perform ABG, and if the parameters were modified, they were transferred to the Intensive Care Department, which functions as an external section. In the ICU, investigations were repeated, or tracheal aspirates were collected in the case of intubated and mechanically ventilated patients.

Severe cases that require non-invasive ventilation or even orotracheal intubation in the Emergency Service were taken directly to the ICU via Pneumology. In this case, the pulmonologist recommended investigations for the ICU.

For pathogens isolated in the ICU, testing for antibiotic resistance was performed using the disk diffusion and minimum inhibitory concentration (MIC) methods. At least 3 to 5 isolated bacterial colonies must be grown from culture to prepare an inoculum suspension necessary for performing the tests.

In our study, the diagnosis of superinfections was made by identifying a pathogenic agent through endotracheal aspirates (64.3%), followed by sputum (16.7%), bronchial aspirates (6%), blood cultures (6%), pleural effusion (2.4%), and nasal tampon (1.2%).

Data were collected as follows: gender, age, COPD diagnosis, stage/classification of the pathology, secondary diagnoses, baseline treatment, general clinical examination, days of hospitalization in the Pulmonology department, and the Intensive Care Unit, investigation methods used to collect biological specimens for confirming or ruling out bacterial superinfection and its results, blood analysis, ventilation mode, progress notes from the Intensive Care Unit, discharge summaries, and discharge status of patients.

Non-invasive ventilation was initially used for patients with moderate to severe COPD exacerbation associated with a pH of ≤7.35 in the first 24 to 48 h, anticipating septic shock, altered neurological status, arterial oxygen pressure (PaO_2_) <45 mmHg, arterial carbon dioxide pressure (PaCO_2_) >70 mmHg, and a pH of ≤7.30. These situations necessitate oropharyngeal intubation and mechanical ventilation. Our patients benefitted from bilevel-positive-airways-pressure (BiPAP) NIV. It supports inspiration, helps decrease respiratory work, and sustain the spontaneous breathing of the patient. The device works as volume-controlled ventilation (VCV) if the patient does not initiate spontaneous respiration. We set up the following:

- Inspiratory pressure (IPAP): 10–18 cmH_2_O
- Expiratory pressure (EPAP): 5–10 cmH_2_O
- Pressure support (PS)—difference between IPAP and EPAP—at least 5 cmH_2_O
- Tidal volume: 6 mL/kg (according to ideal weight)

During the NIV, patients were monitored by performing ABG every 6 h for evaluation of acid-base balance. NIV was replaced with endotracheal intubation and mechanical ventilation if the parameters worsened. The most used mode was pressure support ventilation (PSV), and we set up the following parameters:

- Tidal volume: 6–7 mL/kg (according to ideal weight)
- Respiratory rate: 10–12 respiration/min
- Inspire: Expire ratio: I:E—1:3
- Inspiratory flow: 70–100 L/min
- Positive end-expiratory pressure (PEEP): 4–6 cmH_2_O
- Inspiratory fraction of oxygen (FiO_2_)—55–70%

The study was carried out following the European and national legislation directives and with the principles stated in the Helsinki Declaration. It received approval from the Ethics Committee of the Clinical County Hospital Mureș, Romania, under the number 16779/30.09.2023.

Statistical analysis was performed using IBM SPSS Statistics, version 26.0.0.0 (USA). Data distribution was checked by evaluating kurtosis and skewness parameters, histograms, and Q–Q plots, which were confirmed with the Shapiro–Wilk distribution test and revealed a non-parametric distribution. Quantitative data were expressed as median with minimum–maximum range (median (min–max)), and qualitative data as absolute and relative values (*n* (%)). For statistical differences, Mann–Whitney U and Kruskal–Wallis tests were used for quantitative data, and the Chi-square/Fisher exact test was used for qualitative data. The significance threshold was set at α = 0.05.

## 3. Results

The study involved 84 patients, with an average age of 64 (50–88), with men accounting for 67.90% of the participants. Most patients presented with a COPD diagnosis in higher stages, with 70.2% classified as stage IV according to the GOLD guide. A high proportion of patients included in the study presented with cardiovascular comorbidities (88.1%), 17.9% had a history of pulmonary neoplasia, 7.1% had a history of neoplasia with non-pulmonary localization, 14.3% had a history of diabetes, and 13.1% presented with renal and hepatic comorbidities. Most patients (76.2%) had a history of smoking.

In terms of blood gas analysis, most patients exhibited respiratory acidosis (75%; *n* = 63), reflecting a standard mechanism that occurs during COPD exacerbation and often leads to worse patient outcomes. Overall, 8.3% of patients (*n* = 7) were diagnosed with metabolic acidosis, 7.1% (*n* = 6) with respiratory alkalosis, and 2.4% (*n* = 2) with metabolic alkalosis. Six patients (7.1%) had ABG results without significant modifications.

Regarding the ventilation mechanism, 16% of patients used a simple facial mask, 26% used non-invasive ventilation, and 58% used orotracheal intubation.

**Figure 1:**
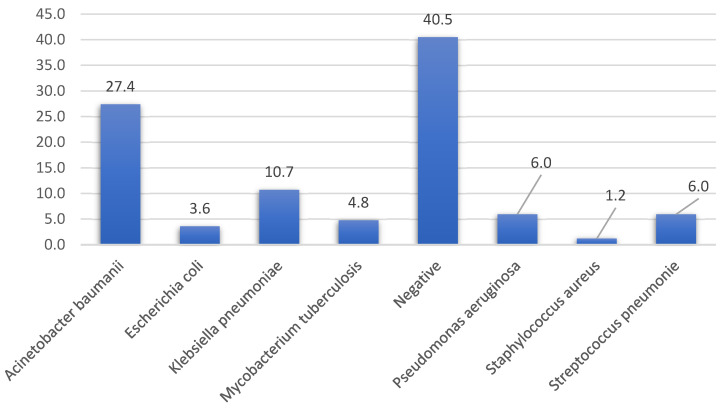
Distribution of pathological agents’ occurrence (%).

Patients had a median white blood cell count of 12.9 × 10^3^/μL (range 5.3–27.8 × 10^3^/μL), and the bacteriological probe was negative in 40.4% of patients. In those with bacterial identification, *Acinetobacter baumannii* infection was more common (27.4%), followed by *Klebsiella pneumoniae* (10.7%). (Figure 1).

Subsequently, patients were divided into groups based on the etiological factor of superinfection. Analysis of laboratory values continued and revealed a significantly higher number of white blood cells in patients superinfected with *E. coli*, with a median of 24.5 × 10^3^/μL. WBC counts were also increased in patients infected with *Pseudomonas aeruginosa* with a median of 16.1 × 10^3^/μL, with *Acinetobacter Baumanii* with a median of 15.1×10^3^/μL, respectively, and *Klebsiella pneumoniae* with an average of 13.4 × 10^3^/μL. (Table 1 + intergroup differences, Table S1—Supplementary File).

C-reactive protein (CRP) values also showed significant differences among the different identified pathogens, with Acinetobacter baumanii registering the highest values (1582), followed by *Mycobacterium tuberculosis* (1223) and Pseudomonas aeruginosa (906). (Table 2 + intergroup differences, Table S2—Supplementary File).

Differences in choices for patient ventilation were also assessed, revealing a significantly (*p* < 0.001) more frequent use of orotracheal intubation in patients with an *Acinetobacter baumanii* and *Staphylococcus aureus* infections (100%)*, Klebsiella pneumoniae* (88.9%), and *Streptococcus Pneumoniae* (80%). Non-invasive ventilation was more frequently used in patients with *Mycobacterium tuberculosis* (75%) and *E. coli* (66.7%) infections. A simple face mask was more frequently used in patients with negative tests for superinfection (Table 3).

Observing the periods of hospital admissions, a significant difference (*p* = 0.04) was noticed, with patients admitted to the ICU spending a median of 5.5 days compared to their median time spent in the Pulmonology Department (2 days), suggesting a high need for intensive care in patients with exacerbated COPD.

The mortality recorded during hospitalization in the analyzed group of patients was 60.7% (*n* = 54 patients). Regarding fatality, *Acinetobacter baumanii* superinfection led to a significantly more frequent rate of death of 39.2% vs. 9.1%, followed by superinfection due to *E. coli,* with a rate of 5.9% versus 0% (Table 4).

## 4. Discussion

The study was meant to illustrate the superinfection scenario for COPD patients, particularly in terms of bacterial profiles, laboratory markers, ventilatory support requirements, and mortality rates correlated with various pathogens. With a study population primarily consisting of older, male patients, a distinctive pattern of superinfection etiologies emerged, shedding light on infection profiles that may inform clinical practice. The data reveal that *Acinetobacter baumannii* was the most frequently observed pathogen among superinfected COPD patients, consistent with other studies that have highlighted its prevalence in hospital-acquired infections and its association with poor outcomes [24]. The high prevalence of multidrug-resistant Acinetobacter baumannii in ICU settings has been highlighted by studies from scientific literature, which is linked to increased ventilation requirements and elevated mortality rates. Furthermore, Pseudomonas aeruginosa superinfection has been linked to prolonged hospital stays and more frequent use of non-invasive ventilation [25]. The multidrug-resistant profile of this pathogen makes it a major challenge to treat, as evidenced by the high mortality observed in this study (30.9%), surpassing all other pathogens analyzed. This mortality trend aligns with recent findings that underscore *A. baumannii*’s role in heightened mortality risk among critically ill COPD patients, especially those requiring intensive respiratory support [26].

Interestingly, our analysis showed a substantial variation in white blood cell counts among patients infected with different pathogens. Patients with *E. coli* infection had the highest median WBC count (24 × 10^3^/μL), followed by *Pseudomonas aeruginosa* and *Acinetobacter baumannii*, suggesting that certain bacterial agents may trigger a stronger inflammatory response. Elevated WBC counts in bacterial infections, particularly those involving Gram-negative pathogens like *E. coli* and *Pseudomonas*, align with the literature on acute inflammatory markers in severe infections [27].

Our findings of elevated WBC count in these patient groups are supported by the fact that Gram-negative bacteria, especially *Escherichia coli* and *Pseudomonas aeruginosa*, cause a more robust systemic inflammatory response [28]. Given these variations, WBC count could potentially serve as an adjunct marker in identifying the bacterial etiology of superinfection in COPD patients. White blood cell counts have consistently been shown to reflect the severity of infectious exacerbations in COPD patients. In the context of bacterial superinfection, elevated WBC levels often signal a heightened immune response and correlate with the severity of an infection [29].

Ventilation requirements differed significantly among pathogen groups. Oro-tracheal intubation (OTI) was more frequently required for patients with *Acinetobacter baumannii* and *Staphylococcus aureus* infections, whereas non-invasive ventilation (NIV) was more commonly used in cases of *Mycobacterium tuberculosis* and *E. coli* infections. This finding aligns with previous studies that have associated certain pathogens, particularly *Acinetobacter* and *Staphylococcus*, with the need for invasive mechanical ventilation due to their virulent respiratory manifestations and association with ventilator-associated pneumonia, as stated in the study conducted by AM Gouda et al. [30]. Conversely, patients with *Mycobacterium tuberculosis* infection demonstrated a preference for non-invasive ventilation, possibly reflecting the distinct disease course and less acute respiratory decompensation typical of tuberculosis in a chronic COPD population [31].

The presence of alkalosis and metabolic acidosis, which are not commonly associated with COPD, could suggest underlying metabolic issues caused by decompensated comorbidities such as renal dysfunction or sepsis-induced changes in acid-base balance. These changes may be justified in the group of study patients by the presence of significant cardiovascular (88.1%) and renal and hepatic (13.1%) comorbidities.

Mortality analysis further underscored the differential impact of pathogens on patient outcomes. *Acinetobacter baumannii* superinfection not only exhibited the highest mortality rate but was also the most significant predictor of fatality, corroborating studies that report similar mortality associations in cases of multidrug-resistant *A. baumannii* infection [26,32,33]. *E. coli* infections, although associated with higher WBC counts, resulted in comparatively lower mortality, indicating that while inflammatory response intensity can indicate disease severity, pathogen-specific virulence factors play an equally crucial role in patient prognosis [34,35].

This study contributes to the understanding of pathogen-specific impacts on the clinical course of COPD patients with superinfection. Future studies should focus on elucidating the mechanisms driving differential WBC responses and ventilatory requirements, potentially incorporating larger cohorts and prospective data collection. Further, given the high mortality associated with certain pathogens, particularly Acinetobacter baumannii, emphasis on targeted preventive measures and early intervention strategies for high-risk patients remains imperative.

### Limitations of the Study

The study’s relatively small cohort of 84 patients limits the generalizability of the findings and reduces the statistical power to detect subtle differences or trends among subgroups. Furthermore, being conducted in a single institution, the results may reflect specific local clinical practices, bacterial resistance patterns, and patient demographics, which could differ from those in other regions or healthcare settings. As a retrospective analysis, the study is subject to inherent biases, including incomplete data collection and reliance on previously documented clinical notes, which may lack uniformity in reporting. Patients with SARS-CoV-2 co-infection or overlap syndromes were excluded, potentially omitting critical cases where mixed pathologies could offer additional insights into ICU outcomes in COPD exacerbations. The study only evaluated in-hospital mortality during ICU stays and did not assess long-term outcomes or mortality post-discharge, leaving the broader impact of superinfection and ventilation strategies open for further investigation. While significant correlations between bacteriological agents and outcomes were identified, the absence of predictive modeling due to limited data limits the ability to use these findings for risk stratification or clinical decision-making. Specific details on ventilation parameters, duration of use, and complications associated with different ventilation modes were not included, which could influence outcomes. Therefore, these findings require prospective studies for validation to establish causal relationships and strengthen their applicability to clinical practice.

## 5. Conclusions

This study highlights the substantial influence of bacterial superinfections on the clinical outcomes of COPD patients admitted to the ICU, with variations in pathogen profiles, inflammatory responses, and ventilation requirements. Notably, *Acinetobacter baumannii* emerged as a critical factor associated with both an increased need for invasive ventilation and elevated mortality, underscoring the challenges posed by multidrug-resistant organisms in these settings. Elevated WBC counts, particularly in *E. coli* and *Pseudomonas aeruginosa* infections, reflect significant inflammatory responses, suggesting potential utility as adjunctive markers for identifying bacterial superinfection in COPD exacerbations. The findings underscore the necessity of targeted preventive and therapeutic strategies, especially for high-risk pathogens like *A. baumannii*, to improve patient prognosis. Future prospective research should aim to clarify the mechanisms driving these pathogen-specific responses and consider interventions tailored to pathogen virulence to optimize treatment approaches for ICU-admitted COPD patients. Additional research should aim to develop rapid diagnostic tools that can aid in the early identification of high-risk bacterial infections and guide timely therapeutic decisions.

## Figures and Tables

**Table 1:** White blood cell counts for different superinfection pathogens.

Bacteriologic Agent	*Acinetobacter baumanii*	*E. coli*	*Klebsiella pneumoniae*	*M. tuberculosis*	*Pseudomonas aeruginosa*	*Staph. aureus*	*Strep. pneumoniae*	*p* ***
WBC count ×10^3^/μL	15.1 (14.3–17.5)	24.5 (19.3–27.8)	13.4 (13.0–13.8)	12.2 (12.1–12.3)	16.1 (14.9–16.7)	12.3 (12.3–12.3)	12.6 (12.4–14.7)	<0.001

*** Kruskal–Wallis test.

**Table 2:** C-reactive protein values for different superinfection pathogens.

Bacteriologic Agent	*Acinetobacter baumanii*	*E. coli*	*Klebsiella pneumoniae*	*M. tuberculosis*	*Pseudomonas aeruginosa*	*Staph. aureus*	*Strep. pneumoniae*	*p* ***
CRP value	1582 (1536–1682)	578 (571–589)	581 (550–730)	1223 (1173–1348)	906 (871–934)	875 (821–954(	605 (569–645)	<0.001

*** Kruskal–Wallis test; CRP—C-reactive protein.

**Table 3:** Ventilation methods for different superinfection pathogens.

Ventilation Mode	*Acinetobacter baumanii*	*E. coli*	*Klebsiella pneumoniae*	*M. tuberculosis*	*Pseudomonas aeruginosa*	*Staph. aureus*	*Strep. pneumoniae*	*p* *
OTI n (%)	23 (100%)	1 (33.3%)	8 (88.9%)	0 (0%)	3 (60%)	1 (100%)	4 (80%)	<0.001
Facial mask n (%)	0 (0%)	0 (0%)	0 (0%)	0 (0%)	0 (0%)	0 (0%)	0 (0%)	
NIV n (%)	0 (0%)	2 (66.7%)	1 (11.1%)	3 (75%)	2 (40%)	0 (0%)	1 (20%)	

* Chi-square test; OTI—orotracheal intubation; NIV—non-invasive ventilation.

**Table 4:** Differences in evolution during hospitalization.

Superinfection Pathogen	Stable State *n* (%)	Deceased *n* (%)	*p* *
*Acinetobacter baumani*	3 (9.1%)	20 (39.2%)	<0.01
*E. coli*	0 (0%)	3 (5.9%)	
*Klebsiella pneumonie*	4 (12.1%)	5 (9.8%)	
*Mycobacterium tuberculosis*	2 (6.1%)	2 (3.9%)	
Negative	22 (66.7%)	12 (23.5%)	
*Pseudomonas aeruginosa*	1 (3%)	4 (7.8%)	
*Staph. aureus*	0 (0%)	1 (2%)	
*Strep. pneumonie*	1 (3%)	4 (7.8%)	

* Chi-square test.

## Data Availability

The dataset is available upon request from the authors.

## Supplementary Materials

The following supporting information can be downloaded at: https://www.mdpi.com/article/10.3390/medicina61040669/s1, Table S1: Intergroup differences for Table 1; Table S2: Intergroup differences for Table 2.
