# Supplementary material for: Analyzing Clinical Parameters and Bacterial Profiles to Uncover the COPD Exacerbations: A Focus on Intensive Care Unit Challenges
Source: Medicina (Kaunas). 2025 Apr 5;61(4):669. doi: 10.3390/medicina61040669 (PMC12029107; doi:10.3390/medicina61040669)
Supplement: Supplementary file 1 [file medicina-61-00669-s001.zip › Table S1.pdf]

**Pathogen**	**p****	**Pathogen**	**p****
Negative-Mycobacterium tuberculosis	0.16	Staphylococcus aureus-Klebsiella pneumoniae	0.69
Negative-Staphylococcus aureus	0.43	Staphylococcus aureus-Acinetobacter baumanii	0.23
Negative-Streptococcus pneumoniae	**<0.01**	Staphylococcus aureus-Pseudomonas aeruginosa	0.19
Negative-Klebsiella pneumoniae	**<0.001**	Staphylococcus aureus-Escherichia coli	0.10
Negative-Acinetobacter baumanii	**<0.001**	Streptococcus pneumoniae-Klebsiella pneumoniae	0.91
Negative-Pseudomonas aeruginosa	**<0.001**	Streptococcus pneumoniae-Acinetobacter baumanii	0.08
Negative-Escherichia coli	**<0.001**	Streptococcus pneumoniae-Pseudomonas aeruginosa	0.09
Mycobacterium tuberculosis-Staphylococcus aureus	0.96	Streptococcus pneumoniae-Escherichia coli	**0.03**
Mycobacterium tuberculosis-Streptococcus pneumoniae	0.54	Klebsiella pneumoniae-Acinetobacter baumanii	**0.04**
Mycobacterium tuberculosis-Klebsiella pneumoniae	0.43	Klebsiella pneumoniae-Pseudomonas aeruginosa	0.07
Mycobacterium tuberculosis-Acinetobacter baumanii	**0.02**	Klebsiella pneumoniae-Escherichia coli	**0.02**
Mycobacterium tuberculosis-Pseudomonas aeruginosa	**0.02**	Acinetobacter baumanii-Pseudomonas aeruginosa	0.65
Mycobacterium tuberculosis-Escherichia coli	**0.01**	Acinetobacter baumanii-Escherichia coli	0.26
Staphylococcus aureus-Streptococcus pneumoniae	0.74	Pseudomonas aeruginosa-Escherichia coli	0.52

****Pairwise comparison p value;
