# Supplementary material for: Analyzing Clinical Parameters and Bacterial Profiles to Uncover the COPD Exacerbations: A Focus on Intensive Care Unit Challenges
Source: Medicina (Kaunas). 2025 Apr 5;61(4):669. doi: 10.3390/medicina61040669 (PMC12029107; doi:10.3390/medicina61040669)
Supplement: Supplementary file 1 [file medicina-61-00669-s001.zip › Table S2.pdf]

Pathogen	p****	Pathogen	p****
**Negative-Staphylococcus aureus**	0.40	Escherichia coli-Streptococcus pneumoniae	0.85
**Negative-Escherichia coli**	0.04	Escherichia coli-Pseudomonas aeruginosa	0.50
**Negative-Klebsiella pneumoniae**	<0.001	Escherichia coli-Mycobacterium tuberculosis	0.37
**Negative-Streptococcus pneumoni**	<0.001	Escherichia coli-Acinetobacter baumanii	0.11
**Negative-Pseudomonas aeruginosa**	<0.001	Klebsiella pneumoniae-Streptococcus pneumoniae	0.83
**Negative-Mycobacterium tuberculosis**	<0.001	Klebsiella pneumoniae-Pseudomonas aeruginosa	0.41
**Negative-Acinetobacter baumanii**	<0.001	Klebsiella pneumoniae-Mycobacterium tuberculosis	0.27
**Staphylococcus aureus-Escherichia coli**	0.78	Klebsiella pneumoniae-Acinetobacter baumanii	<0.01
**Staphylococcus aureus-Klebsiella pneumoniae**	0.74	Streptococcus pneumoniae-Pseudomonas aeruginosa	0.58
**Staphylococcus aureus-Streptococcus pneumoniae**	0.67	Streptococcus pneumoniae-Mycobacterium tuberculosis	0.41
**Staphylococcus aureus-Pseudomonas aeruginosa**	0.46	Streptococcus pneumoniae-Acinetobacter baumanii	0.09
**Staphylococcus aureus-Mycobacterium tuberculosis**	0.36	Pseudomonas aeruginosa-Mycobacterium tuberculosis	0.76
**Staphylococcus aureus-Acinetobacter baumanii**	0.20	Pseudomonas aeruginosa-Acinetobacter baumanii	0.32
**Escherichia coli-Klebsiella pneumoniae**	0.97	Mycobacterium tuberculosis-Acinetobacter baumanii	0.59

****Pairwise comparison p value;
